# Evaluation of a method based on synthetic data inserted into raw data prior to reconstruction for the assessment of PET scanners

**DOI:** 10.1186/s40658-022-00496-6

**Published:** 2022-10-01

**Authors:** Quentin Maronnier, Frédéric Courbon, Olivier Caselles

**Affiliations:** Medical Physics Department, Institut Claudius Regaud, Toulouse, France

**Keywords:** Positron emission tomography, Performance, Methods, Phantom, Experiment, Simulation

## Abstract

**Background:**

Performance assessment of positron emission tomography (PET) scanners is crucial to guide clinical practice with efficiency. Even though clinical data are the final target, their use to characterize systems response is constrained by the lack of ground truth. Phantom tests overcome this limitation by controlling the object of study, but remain simple and are not representative of patient complexity. The objective of this study is to evaluate the accuracy of a simulation method using synthetic spheres inserted into acquired raw data prior to reconstruction, simulating multiple scenarios in comparison with equivalent physical experiments.

**Methods:**

We defined our experimental framework using the National Electrical Manufacturers Association NU-2 2018 Image Quality standard, but replaced the standard sphere set with more appropriate sizes (4, 5, 6, 8, 10 and 13 mm) better suited to current PET scanner performance. Four experiments, with different spheres-to-background ratios (2:1, 4:1, 6:1 and 8:1), were performed. An additional dataset was acquired with a radioactive background but no activity within the spheres (water only) to establish a baseline. Then, we artificially simulated radioactive spheres to reproduce other experiments using synthetic data inserted into the original sinogram. Images were reconstructed following standard guidelines using ordered subset expectation maximization algorithm along with a Bayesian penalized likelihood algorithm. We first visually compared experimental and simulated images. Afterward, we measured the activity concentration values into the spheres to calculate the mean and maximum recovery coefficients (RC_mean_ and RC_max_) which we used in a quantitative analysis.

**Results:**

No significant visual differences were identified between experimental and simulated series. Mann–Whitney *U* tests comparing simulated and experimental distributions showed no statistical differences for both RC_mean_ (*P* value = 0.611) and RC_max_ (*P* value = 0.720). Spearman tests revealed high correlation for RC_mean_ (*ρ* = 0.974, *P* value < 0.001) and RC_max_ (*ρ* = 0.974, *P* value < 0.001) between both datasets. From Bland–Altman plots, we highlighted slight shifts in RC_mean_ and RC_max_ of, respectively, 2.1 ± 16.9% and 3.3 ± 22.3%.

**Conclusions:**

We evaluated the efficiency of our hybrid method in faithfully mimicking practical situations producing satisfactory results compared to equivalent experimental data.

**Supplementary Information:**

The online version contains supplementary material available at 10.1186/s40658-022-00496-6.

## Background

Since the introduction of the first positron emission tomography (PET) scanners, increasingly higher sensitivity and improved spatial and timing resolution have become available thanks to hardware improvement (scintillator crystal, photodetector, electronics) and software development (reconstruction and image analysis) [1]. Overall performances of PET devices have thereby been greatly increased. Hence, performance evaluation is crucial to guide clinical practice with efficiency. Even though clinical data are the final target, their use to characterize systems response is constrained by the lack of ground truth. Therefore, the assessment of their performances is achieved through tests based on standards that were defined by scientific experts from national and international authorities such as the National Electrical Manufacturers Association (NEMA) and the International Electrotechnical Commission (IEC) [2, 3]. These procedures are relevant for investigating and benchmarking the scanners using a standard object whose parameters are controlled [4], but are often not appropriate to current clinical challenges such as the detectability of subcentimeter lesions [5, 6] or to patient complexity [7]. These objects, commonly named phantoms, consist of relatively simple geometrical objects, fillable with radioactive aqueous solutions. Phantom preparation and acquisition require material, radiotracer and scanner availability, which can be complex to schedule besides clinical practice. Thus, it is difficult to generate large samples of experimental data due to limited resources and accessibility.

Simulation provides an alternative method to the use of physical data through computational modeling. The most realistic is particle-tracking-based simulation, which is generically referred to as Monte Carlo method [8, 9]. In medical imaging, the SimSET package uses Monte Carlo techniques to model the physical processes and instrumentation, in particular in PET imaging [10]. A major limitation in particle-tracking simulation is the significant computation time which limits the generation of large datasets. Analytical simulation is another method that models the average probability of photon interactions instead of individual photon tracking. Therefore, it significantly improves computational cost and can generate large datasets promptly [10–15].

By combining physical (phantom and patient) data with fast analytical simulation, we could quickly generate significant sets of imaging configurations to explore for instance lesion detection [14, 15]. Hence, it would make it possible to generate a large number of different datasets from a single physical sample [13–15]. The data insertion simulation method consists in embedding synthetic information with known characteristics such as location, volume, shape and activity into pre-acquired data using system modeling. Technically, the data insertion simulation method relies on the forward projection through the scanner model of the synthetic spheres into simulated sinogram. It is then summed to the original sinogram to obtain new raw data for the reconstruction of simulated datasets. The purpose of such simulation method is to link practicality and ground truth.

In order to design a hybrid method, Juma [10] used and compared two simulation techniques, SimSET and a forward projector, that generates lesion sinogram estimating events from an estimated activity map. He considered the analytical simulation as the most appropriate method, taking into account simulation times, technical limitations related to file formats and the opportunity to easily simulate time-of-flight (TOF). In a recent study, Gabrani-Juma et al. [14] contrasted results obtained with analytical simulation using a digital phantom to NEMA image quality (IQ) results and observed that the simulated spheres showed a systematic overestimation of about 20% compared to the physical spheres for sizes smaller than 22 mm in diameter. However, the digital phantom did not have the same geometry and volume as the physical NEMA body phantom. Hence, it is difficult to conclude whether this oversight came from the simulation or structural differences of the study object.

We have similar analytical simulation and a reconstruction toolbox for remote reconstruction provided by the manufacturer through a research collaboration. Our final goal is to use it on clinical data to create a scalable ground truth responding to the clinical need. Prior to that, we currently focused on its validation first in contrast to experimental data defined as a reference. In this study, we aimed to introduce a hybrid simulation method and to evaluate its accuracy in multiple scenarios in comparison with equivalent physical experiments.

## Methods

### PET–CT system

All the experiments were performed on the Discovery MI 5-ring positron emission tomography-computed tomography (PET–CT) digital system (General Electric Healthcare, Chicago, IL, USA). This PET–CT device is combining TOF, high resolution and high sensitivity, which improve overall image quality by reducing the noise in the reconstructed images and enhance lesion detection [16]. There are several reconstruction algorithms available to produce images from the acquired raw data such as the common ordered subset expectation maximization (OSEM). A more recent Bayesian penalized likelihood (BPL) algorithm, gives access to a regularization parameter *β* that allows to reduce image noise through each iteration [17, 18]. Results from the NEMA NU2-2012 standard performance tests for this configuration of the device have been published [19]. The evaluation of its performances is important but is not sufficiently discriminating compared to its capabilities such as subcentimeter lesion detection. We performed several experiments based on the NEMA NU-2 2018 IQ performance standard in which we adapted the set of fillable spheres for challenging smaller sizes.

### Phantoms experiments

**Fig. 1:**
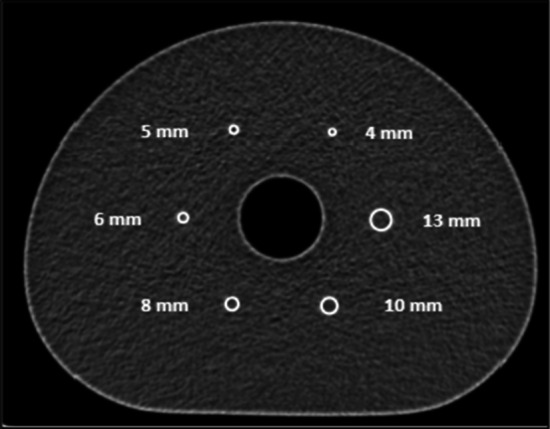
Central section of the spheres from CT images

We opted for the NEMA Image Quality NU-2 2018 test because it aims to simulate a PET–CT whole-body clinical use case [2]. We used the body phantom with its lung insert, but replaced the standard fillable spheres by another set with smaller internal diameters of, respectively, 4, 5, 6, 8, 10 and 13 mm (Data Spectrum Corporation, Durham, NC, USA). A central axial section of the phantom is shown in Fig. 1.

As required by the standard, we placed a scatter phantom on the cradle outside the scanner field of view. The filling of the phantoms, their positioning and their acquisitions on the examination bed were carried out according to the standard recommendation. Five distinct experiments were performed corresponding to five concentrations of ^18^F leading to five spheres-to-background ratios (SBR) of approximately 2:1, 4:1, 6:1, 8:1 and finally 0:1 (water only within the spheres) as a baseline for data insertion. The radioactive concentration of the background was nearly the same for all the experiments in order to get rather the same total activity within the body phantom. Details of the filling levels in the different compartments of the scanned phantom are available in Table 1.

**Table 1:** Activity concentration (kBq/mL) within the background and spheres of the body phantom for each series

	Activity concentration (AC) (kBq/mL)		Resulting SBR
	Background	Spheres	
Experiment SBR 2:1	5.32	11.03	2.07
Experiment SBR 4:1	5.44	21.38	3.93
Experiment SBR 6:1	5.50	33.19	6.03
Experiment SBR 8:1	5.32	42.42	7.97
Experiment SBR 0:1	5.52	0.00	0.00

We chose to conduct this study with OSEM and BPL algorithms because the former is part of the standard procedure and the latter is used in clinical routine for reporting by the physicians in our institution. It allowed us to compare the impact of the reconstruction algorithms using our method. Images were obtained using acquisition and reconstruction parameters detailed in Table 2.

**Table 2:** Acquisition and reconstruction parameters

Acquisitions durations (s):	323/334/346
Matrix size:	384 × 384
FOV (mm):	400
Slice thickness (mm):	2.8
Voxel dimensions (mm):	1.042 × 1.042 × 2.8
Voxel volume (mm^3^):	3.04
*Algorithm 1*	
Reconstruction algorithm:	OSEM
TOF:	Yes
Iterations:	4
Subsets:	34
Filter (FWHM):	2
Z-filter:	None
Corrections:	Attenuation, scatter, randoms
Point spread function (PSF) modeling:	No
*Algorithm 2*	
Reconstruction algorithm:	BPL
TOF:	Yes
Beta (*β*):	20
Corrections:	Attenuation, scatter, randoms
Point spread function (PSF) modeling:	Yes

### Simulation method

The simulation method consists of inserting synthetic information with known characteristics, such as location, volume and activity, into pre-acquired raw data using system modeling. Finally, modified raw data are reconstructed into simulated axial images [13–15].

Offline data insertion and image reconstruction were achieved using a reconstruction research toolbox (Duetto v02.13, General Electric Healthcare, Chicago, IL, USA).

Beforehand, we exported the raw data and computed tomography attenuation correction (CTAC) images from the scanner to a dedicated high-performance research workstation Z8 (Hewlett-Packard, Palo Alto, CA, USA) where modeling, simulation and reconstruction were executed using MATLAB R2018b (The MathWorks Inc., Natick, MA, USA).

#### Modeling

First, we determined the spatial coordinates of the center of each physical sphere from the CT images. We modeled the spheres according to the internal diameter of the physical set using a coded MATLAB function. Spatial coordinates and internal diameter were used as input data in the function as a complement to the exam dimensions, the voxel dimensions and a factor value for upsampling. Due to the limitation of a finite sampled voxel size, a binary mask with the current image resolution would be insufficient to represent a real-world sphere. In addition, the modeling step presents a constraint we have to consider for the data insertion and reconstruction afterward. The synthetic information inserted into the pre-acquired data should have commonalities in terms of reconstruction and acquisition parameters, such as matrix size or slice thickness. These parameters are crucial as they impact the size of the voxels, which must be identical to the original examination.

In this study, the sphere mask was generated by the following steps:

1. Creation of an empty matrix (full of zero) whose dimensions correspond to the dimensions of the original exam;
2. Upsampling the image grid with a given factor in x, y and z dimensions (16 here);
3. Determining a binary sphere mask in the upsampled resolution according to the sphere diameter and sub-voxels sizes;
4. Calculating the mean value of the sub-voxels contained in the same voxel prior to the downsampling of the image grid to its original size.

A schematic illustration of these steps can be found in Additional file 1: Fig. S1.

As the sphere mask was generated by using the mean values, the voxel value in the mask would no longer be binary, but would contain floating point values between zero and one, and could best represent a sphere used in a typical physical phantom. In Table 3, we contrasted the volume of each physical sphere with the calculated volume of the corresponding synthetic sphere using the number of voxels composing it, their value (between 0 and 1) and the theoretical volume of a voxel (available in Table 2).

**Table 3:** Comparison between physical and synthetic internal diameter/volume for each sphere size

Physical diameter (mm)	Physical volume (mm^3^)	Synthetic diameter (mm)	Synthetic volume (mm^3^)
12.43	1005.57	12.43	1004.9
9.89	506.51	9.88	505.8
7.86	254.25	7.85	253.64
6.23	126.61	6.22	126.46
4.95	63.51	4.94	63.52
3.95	32.27	3.95	32.23

In the last step, we had to determine the activity concentration (AC) (Bq/mL) that would be inserted inside the synthetic spheres. To achieve that, we drew 12 Volumes-of-Interest (VOI) distributed in the phantom background. We extracted two specific tags from the DICOM images header: RescaleSlope and RescaleIntercept, which were used to convert image intensities into activity concentrations (Bq/mL). These tags had varying values depending on the slice. Equation (1) represents this step for each image numbered S (Slice number).

1 $${\text{AC}} \left( {\text{Bq/mL}} \right) = {\text{DICOM}}\;{\text{Intensity}} \left( S \right)*{\text{RescaleSlope}} \left( S \right) + {\text{RescaleIntercept}} \left( S \right)$$

Then, we calculated the average AC value within each VOI and afterward determined the average AC value across all VOIs and defined an AC baseline (Bq/mL) to generate insertions directly related to the initial experiment. Finally, we multiplied the sphere mask by the AC baseline value and the exact value of each SBR to simulate the four synthetic sets of images mimicking the acquired experiments.

#### Data insertion

Once these preliminary steps have been completed, we conducted the generation of modified raw data using specific functions provided by the manufacturer. The insertion process relies on the forward projection through the scanner model of the synthetic spheres into simulated sinogram. It is part of the reconstruction research toolbox. During this process, scanner and phantoms effects (geometric efficiency, detector efficiency variations, resolution and attenuation) are applied using raw data and CTAC images from the original acquisition [14, 15]. Poisson noise realization is applied to estimate noise of the inserted data. It is based on the counts per second per unit volume of inserted spheres in the image domain prior to the forward projection. Ultimately, original and simulated sinograms were summed to obtain new raw data used for the reconstruction of the simulated datasets.

#### Image reconstruction

Offline image reconstruction was achieved using the reconstruction research toolbox. Those reconstructions are numerically equivalent to the reconstruction processing available on the PET console. Reconstructed DICOM images are finally uploaded to the interpretation console database for analysis.

**Fig. 2:**
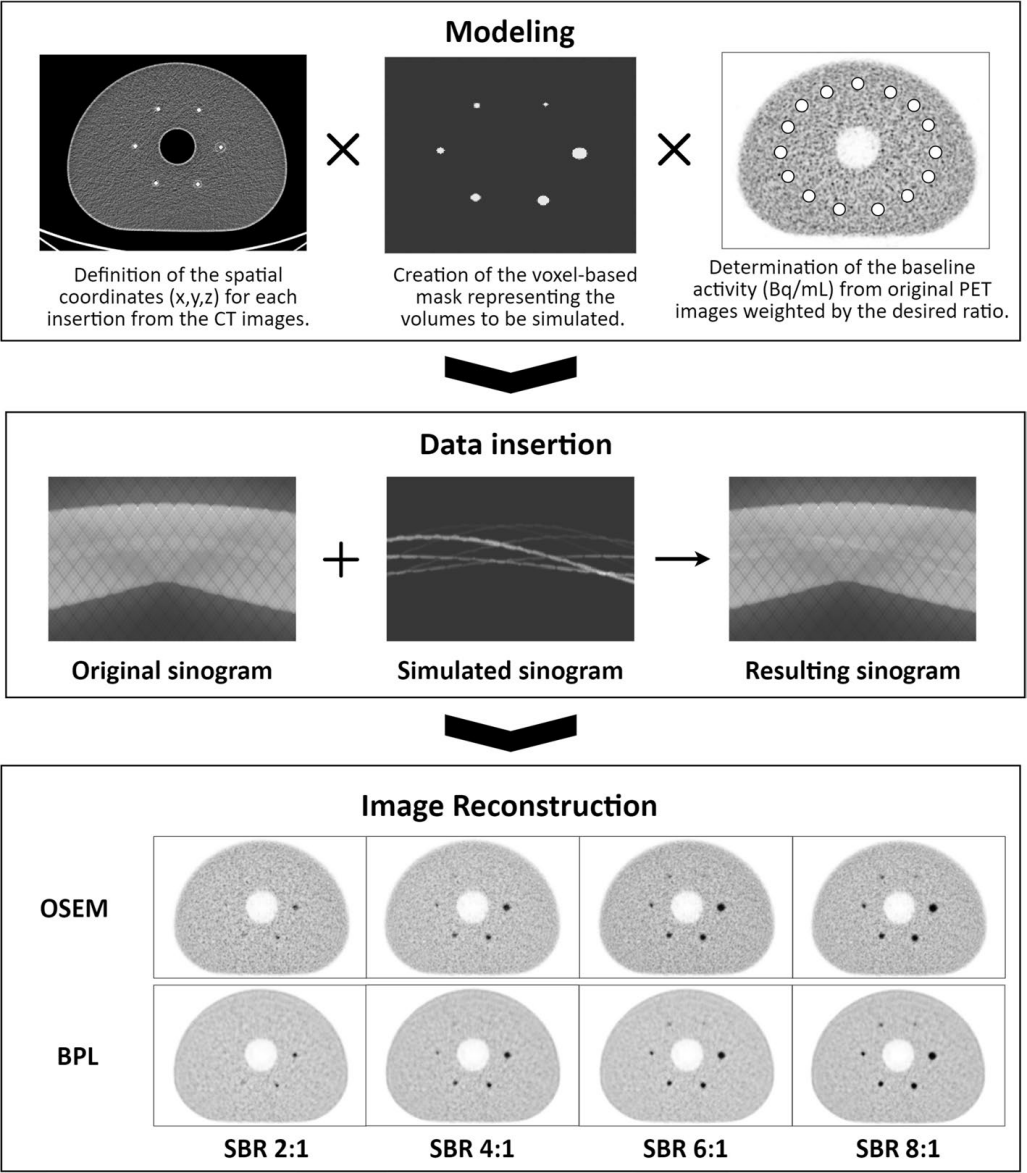
Workflow of the data insertion process

The entire simulation process is illustrated in Fig. 2.

### Images and data analysis

A statistical data analysis was conducted in order to assess the similarity between the simulated and the experimental data considered as the reference. We first assessed the visual equivalence of the simulated images compared to the experimental results. For each set of acquisitions, we had three sets of images, equivalent in terms of total counts, which we averaged to obtain a single frame. Visual inspection was performed on the central slice allowing to get all the spheres in the same plane. Afterward, we also performed a quantitative analysis using the image interpretation software PETVCAR^®^ on the AWServer client console (General Electric Healthcare, Chicago, IL, USA). To perform this analysis, we placed, for each set of images, 6 spherical VOIs centered on each sphere. The volume of each VOI aimed at reproducing the real internal volume of the corresponding spheres and was identical for experimental and simulated series. This study focused on the maximum and mean activity concentrations (kBq/mL) within the spheres. As required by the NEMA standard, each experimental and simulated series consisted of three successive acquisitions which were analyzed individually, following the same measurement conditions, and then averaged to improve the reproducibility of the results. Finally, taking into account all ratios studied and all sphere sizes, we obtained 48 measurements of mean and maximum activity concentrations (kBq/mL), AC_mean_ and AC_max_, for both experimental and simulated series (6 spheres × 2 algorithms × 4 SBR). We then calculated the mean and maximum recovery coefficient, RC_mean_ and RC_max,_ dividing AC_mean_ and AC_max_ by the theoretical activity concentrations deducted from phantom preparation (2).

2 $${\text{RC}}_{{\text{mean/max}}} = \frac{{{\text{AC}}_{{\text{mean/max}}} \left( {\text{kBq/mL}} \right)}}{{{\text{AC}}_{{{\text{theoretical}}}} \left( {\text{kBq/mL}} \right)}}$$

As the background activity might vary from one experiment to another, even if the SBR was the same, we performed this calculation in order to normalize the different datasets and avoid discrepancies between reiterations (Table 1). Simulated and experimental averaged recovery coefficient (RC) were compared considering entire datasets and reconstruction algorithms datasets. First, we carried out a nonparametric Mann–Whitney test to highlight any differences in RC_mean_ and RC_max_ distributions between the experimental and simulated data. Then, we performed a Spearman test [20] to estimate their crossed correlation. Moreover, we verified the normality of the distribution of differences between experimental and simulated quantitative data using D’Agostino–Pearson test [21], in order to further refine the statistical analysis creating Bland–Altman plots [22, 23]. These tests were carried out on all the experimental and simulated data and subsequently repeated for each algorithm. The criterion for the significant difference was *p* < 0.05 for the Mann–Whitney and D’Agostino–Pearson tests. A strong correlation was identified by *p* < 0.05 and *ρ* > 0.8.

## Results

### Visual comparison

**Fig. 3:**
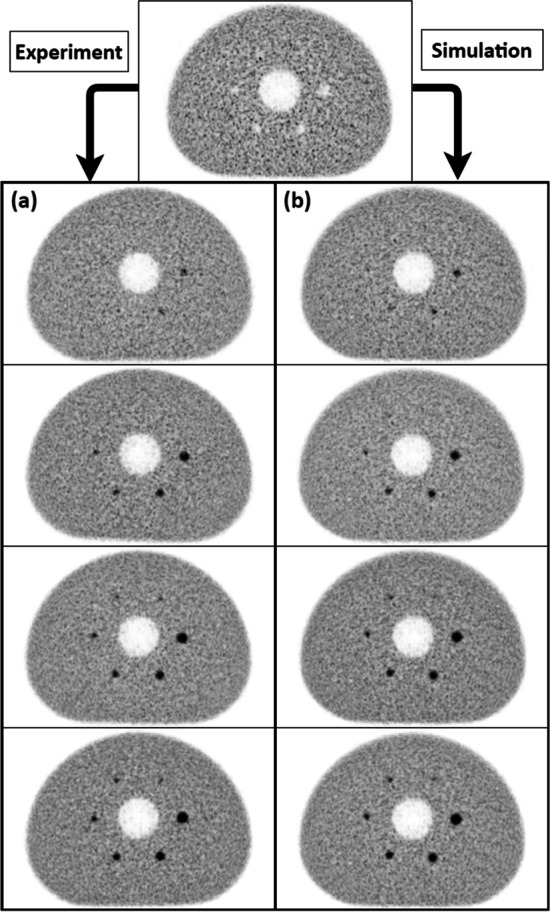
Visual comparison of OSEM averaged reconstructed images: **a** Experimental series (from top to bottom, respectively, SBR 2:1, 4:1, 6:1 and 8:1). **b** Simulated series (from top to bottom, respectively, SBR 2:1, 4:1, 6:1 and 8:1)

**Fig. 4:**
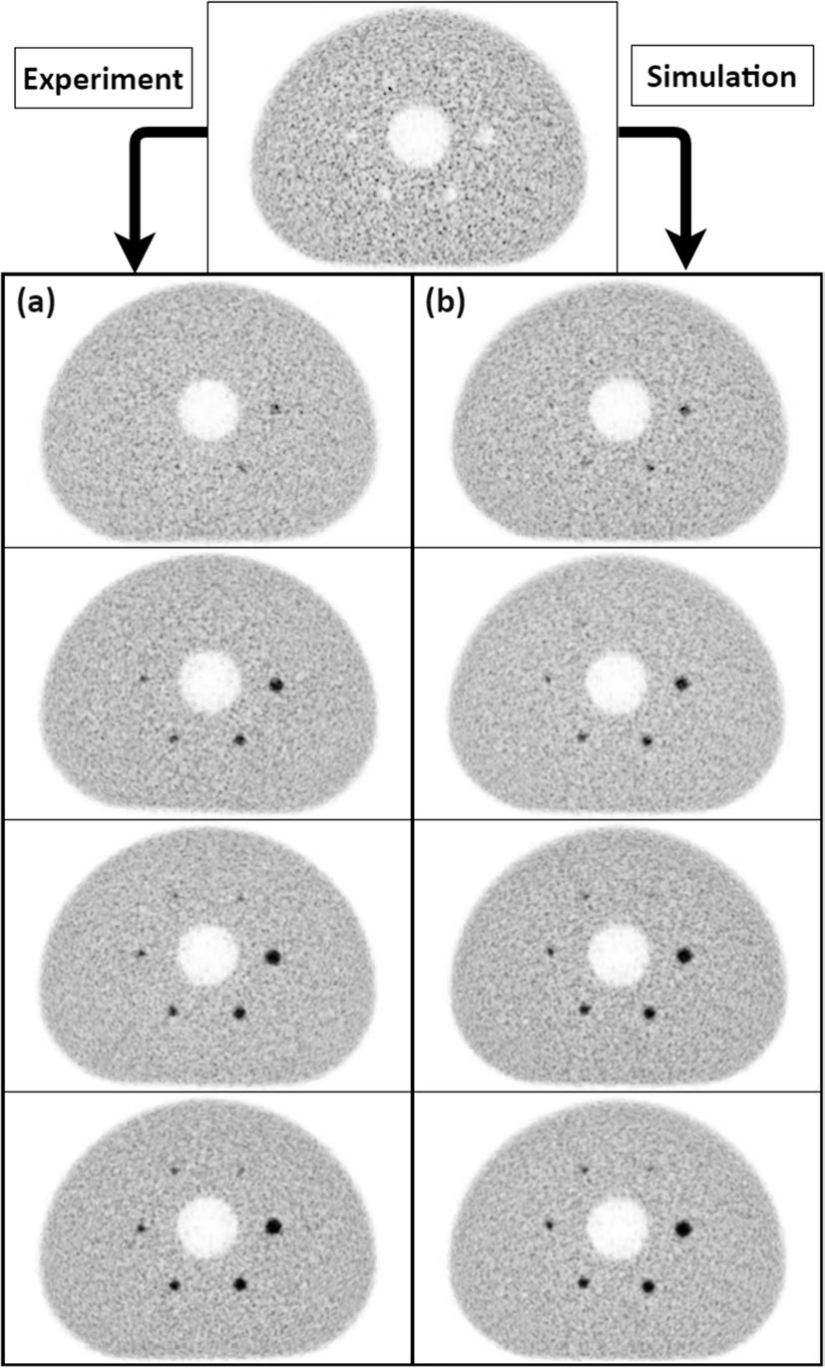
Visual comparison of BPL averaged reconstructed images: **a** Experimental series (from top to bottom, respectively, SBR 2:1, 4:1, 6:1 and 8:1). **b** Simulated series (from top to bottom, respectively, SBR 2:1, 4:1, 6:1 and 8:1)

As expected, due to the creation process of the simulated images, the visual aspect of the background in both experimental and inserted data was similar. Considering the spheres, the same objects could be visualized in both series. However, we could spot slight differences at the PET performance limits for low contrast and small targets, such as the 8 mm sphere at SBR 2:1 and 5 mm at SBR 4:1. This observation was reported for both OSEM and BPL algorithms. An overview of the images is available in Figs. 3 and 4, respectively, for OSEM and BPL reconstruction.

### RC comparison

The results provided by the quantitative analysis for both experimental and simulated data are expressed as average and standard deviation (SD) for each SBR (column) and sphere size (row) in Tables 4 and 5.

**Table 4:** Experimental and simulated RC values from OSEM algorithm for each SBR and sphere size

	OSEM							
	Experimental series				Simulated series			
	SBR 2:1	SBR 4:1	SBR 6:1	SBR 8:1	SBR 2:1	SBR 4:1	SBR 6:1	SBR 8:1
Sphere	Mean recovery coefficient: Average ± SD							
13 mm	0.67 ± 0.04	0.65 ± 0.02	0.64 ± 0.01	0.60 ± 0.01	0.71 ± 0.03	0.66 ± 0.02	0.64 ± 0.01	0.63 ± 0.01
10 mm	0.65 ± 0.03	0.58 ± 0.02	0.57 ± 0.02	0.56 ± 0.01	0.69 ± 0.04	0.63 ± 0.02	0.61 ± 0.02	0.60 ± 0.01
8 mm	0.56 ± 0.04	0.45 ± 0.03	0.47 ± 0.01	0.45 ± 0.01	0.58 ± 0.04	0.53 ± 0.03	0.51 ± 0.03	0.50 ± 0.02
6 mm	0.43 ± 0.06	0.46 ± 0.01	0.40 ± 0.01	0.36 ± 0.03	0.49 ± 0.05	0.43 ± 0.03	0.40 ± 0.02	0.38 ± 0.02
5 mm	0.42 ± 0.08	0.32 ± 0.06	0.30 ± 0.02	0.29 ± 0.05	0.45 ± 0.06	0.35 ± 0.04	0.31 ± 0.02	0.30 ± 0.02
4 mm	0.42 ± 0.14	0.29 ± 0.03	0.32 ± 0.05	0.24 ± 0.05	0.40 ± 0.06	0.31 ± 0.06	0.26 ± 0.05	0.23 ± 0.04
Sphere	Maximum recovery coefficient: Average ± SD							
13 mm	1.67 ± 0.29	1.22 ± 0.02	1.12 ± 0.02	0.99 ± 0.03	1.49 ± 0.08	1.26 ± 0.04	1.16 ± 0.04	1.11 ± 0.03
10 mm	1.46 ± 0.24	1.11 ± 0.06	1.05 ± 0.11	0.93 ± 0.06	1.49 ± 0.17	1.27 ± 0.13	1.16 ± 0.11	1.10 ± 0.09
8 mm	1.11 ± 0.08	0.83 ± 0.15	0.83 ± 0.03	0.87 ± 0.06	1.08 ± 0.23	0.92 ± 0.03	0.84 ± 0.04	0.75 ± 0.10
6 mm	0.70 ± 0.04	0.79 ± 0.13	0.70 ± 0.13	0.63 ± 0.03	0.98 ± 0.07	0.81 ± 0.03	0.74 ± 0.04	0.65 ± 0.11
5 mm	0.67 ± 0.23	0.48 ± 0.08	0.47 ± 0.05	0.42 ± 0.10	0.72 ± 0.06	0.55 ± 0.05	0.49 ± 0.06	0.44 ± 0.09
4 mm	0.55 ± 0.14	0.38 ± 0.07	0.41 ± 0.11	0.30 ± 0.08	0.62 ± 0.16	0.42 ± 0.08	0.34 ± 0.07	0.31 ± 0.05

**Table 5:** Experimental and simulated RC values from BPL algorithm for each SBR and sphere size

	BPL							
	Experimental series				Simulated series			
	SBR 2:1	SBR 4:1	SBR 6:1	SBR 8:1	SBR 2:1	SBR 4:1	SBR 6:1	SBR 8:1
Sphere	Mean recovery coefficient: Average ± SD							
13 mm	0.70 ± 0.04	0.73 ± 0.01	0.73 ± 0.01	0.70 ± 0.01	0.75 ± 0.04	0.73 ± 0.02	0.74 ± 0.01	0.74 ± 0.01
10 mm	0.69 ± 0.03	0.69 ± 0.01	0.71 ± 0.01	0.70 ± 0.01	0.73 ± 0.05	0.72 ± 0.02	0.74 ± 0.02	0.73 ± 0.01
8 mm	0.59 ± 0.05	0.54 ± 0.05	0.61 ± 0.02	0.59 ± 0.00	0.59 ± 0.03	0.61 ± 0.02	0.65 ± 0.03	0.65 ± 0.01
6 mm	0.44 ± 0.06	0.49 ± 0.02	0.51 ± 0.03	0.47 ± 0.08	0.44 ± 0.03	0.46 ± 0.03	0.51 ± 0.06	0.52 ± 0.02
5 mm	0.41 ± 0.09	0.34 ± 0.05	0.33 ± 0.04	0.35 ± 0.06	0.35 ± 0.07	0.28 ± 0.05	0.29 ± 0.03	0.30 ± 0.04
4 mm	0.40 ± 0.07	0.27 ± 0.03	0.31 ± 0.04	0.28 ± 0.05	0.40 ± 0.07	0.30 ± 0.05	0.27 ± 0.05	0.25 ± 0.04
Sphere	Maximum recovery coefficient: Average ± SD							
13 mm	1.65 ± 0.30	1.65 ± 0.38	1.37 ± 0.03	1.39 ± 0.12	1.78 ± 0.13	1.62 ± 0.06	1.51 ± 0.05	1.43 ± 0.07
10 mm	1.57 ± 0.31	1.62 ± 0.20	1.46 ± 0.05	1.40 ± 0.09	1.63 ± 0.21	1.77 ± 0.18	1.78 ± 0.19	1.59 ± 0.13
8 mm	1.06 ± 0.23	1.06 ± 0.30	1.28 ± 0.10	1.55 ± 0.42	1.05 ± 0.17	1.18 ± 0.27	1.46 ± 0.18	1.47 ± 0.06
6 mm	0.64 ± 0.12	0.85 ± 0.06	1.06 ± 0.28	1.02 ± 0.18	0.75 ± 0.04	0.80 ± 0.06	1.03 ± 0.08	1.11 ± 0.11
5 mm	0.54 ± 0.15	0.50 ± 0.01	0.45 ± 0.08	0.51 ± 0.06	0.49 ± 0.09	0.40 ± 0.05	0.42 ± 0.01	0.46 ± 0.02
4 mm	0.49 ± 0.06	0.40 ± 0.04	0.40 ± 0.07	0.36 ± 0.08	0.47 ± 0.12	0.37 ± 0.06	0.33 ± 0.07	0.31 ± 0.05

We highlighted RC_mean_ relative errors inferior to 20% for all configurations, and the same for RC_max_ except for 6 mm sphere at SBR 2:1 (OSEM), which was not visible on the image and gave a relative error of 39%. Otherwise, neglecting this extreme value, the RC_max_ relative error was within 23%. Considering the impact of the reconstruction algorithm, the OSEM algorithm gave the largest relative error differences for both RC_mean_ and RC_max_.

**Fig. 5:**
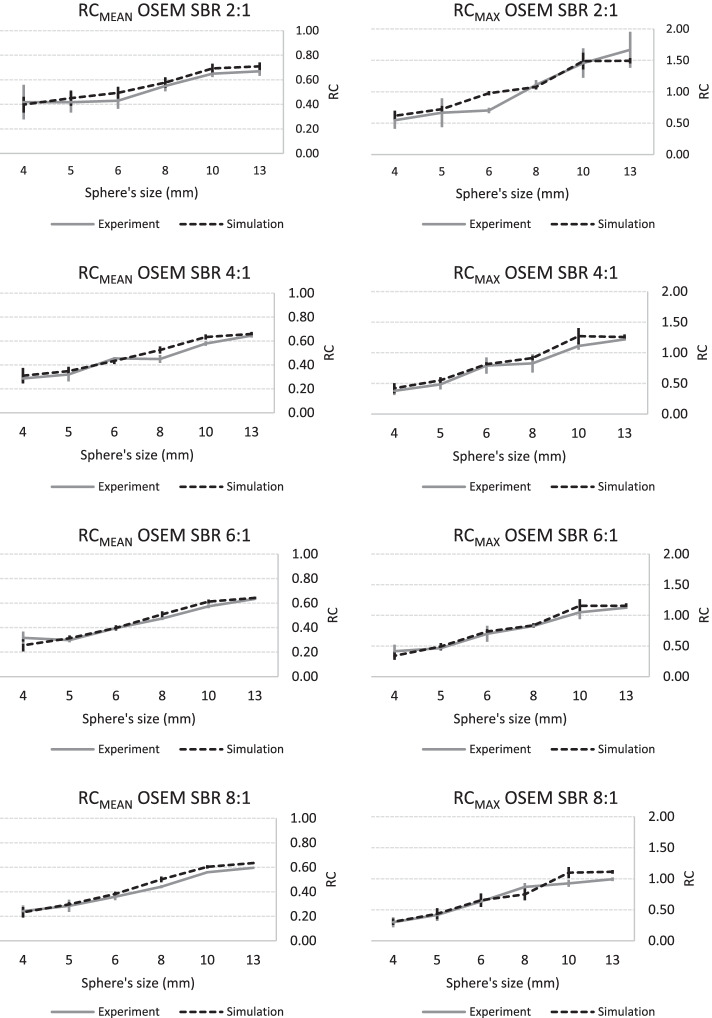
Experimental and simulated RC curves using OSEM algorithm as a function of sphere size: RCmean and RCmax curves are arranged in two columns (left and right, respectively) and SBR ordered in rows (from top to bottom: 2:1, 4:1, 6:1 and 8:1)

A graphical representation of these data is available in Figs. 5 (OSEM) and 6 (BPL), which indicates similar trends for the variation of RC_mean_ and RC_max_ versus sphere size for both synthetic and physical spheres whatever the SBR.

**Fig. 6:**
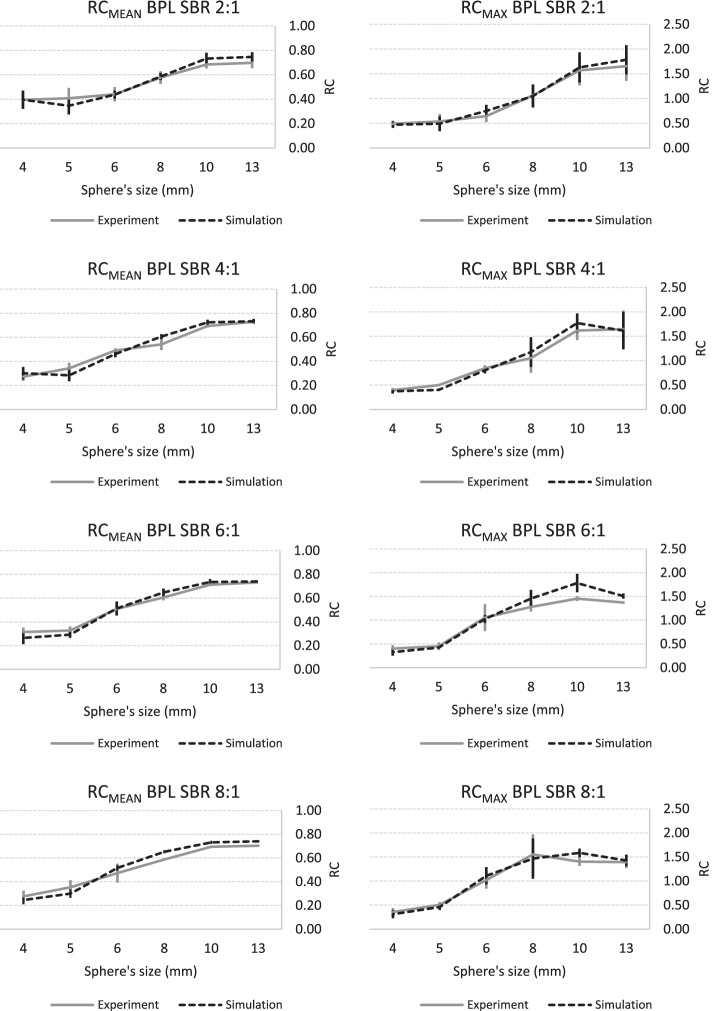
Experimental and simulated RC curves using BPL algorithm as a function of sphere size: RCmean and RCmax curves are arranged in two columns (left and right, respectively) and SBR ordered in rows (from top to bottom: 2:1, 4:1, 6:1 and 8:1)

**Fig. 7:**
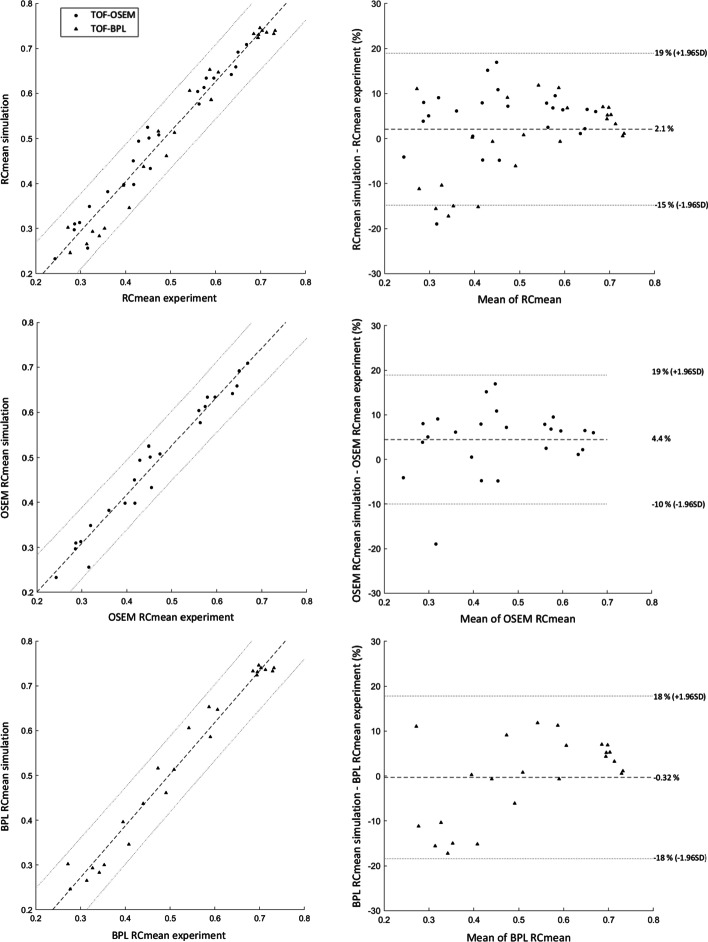
Correlation curve (left) and Bland–Altman plot (right) of RC_mean_ data: Different configurations are ordered in rows (from top to bottom: whole RC_mean_, OSEM RC_mean_ and BPL RC_mean_)

**Fig. 8:**
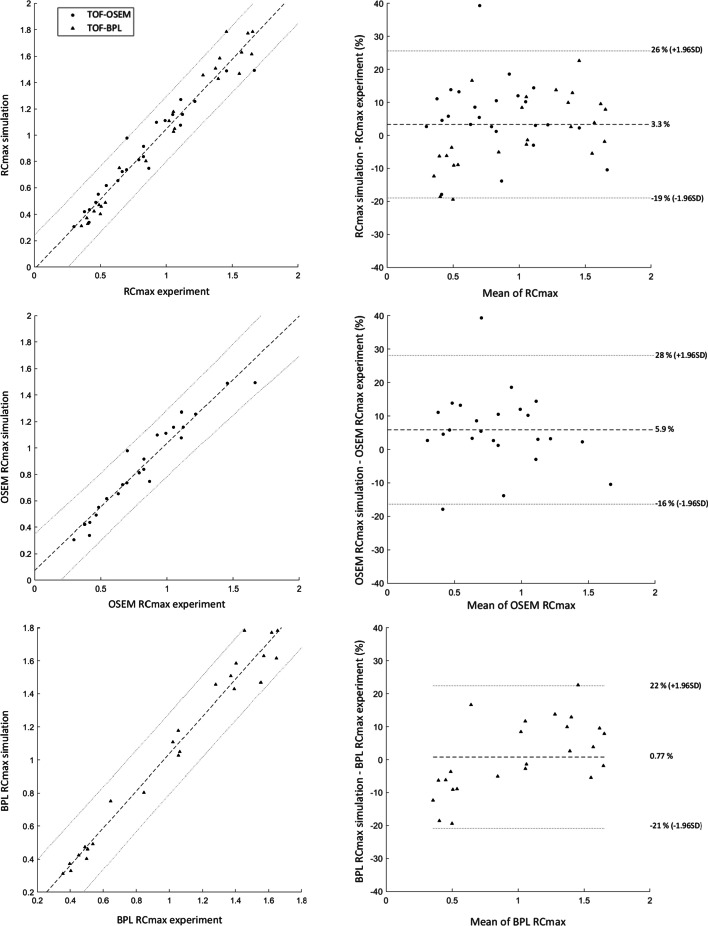
Correlation curves (left) and Bland–Altman plots (right) of RC_max_ data: Different configurations are ordered in rows (from top to bottom: whole RC_max_, OSEM RC_max_ and BPL RC_max_)

The Mann–Whitney test showed no statistical differences between experimental and simulated RC values (both RC_mean_ and RC_max_). This was also confirmed when we studied the impact of the reconstruction algorithm on RC values, despite a higher value for the BPL algorithm. Spearman tests revealed a strong correlation for all datasets (*ρ* > 0.950). From Bland–Altman plots, we determined the mean differences and agreement intervals (within 95% of the differences). Considering all data, we obtained results for RC_mean_ and RC_max_ of 2.1 ± 16.9% and 3.3 ± 22.3%, respectively. This analysis showed a small difference between OSEM and BPL for both RC_mean_ (respectively, 4.4 ± 14.5% vs. − 0.32 ± 18.1%) and RC_max_ (5.9 ± 22.2% vs. 0.77 ± 21.6%). All the results of the statistical analysis are presented in Table 6. Figures 7 and 8 show the correlation curves and Bland–Altman plots for RC_mean_ and RC_max_, respectively.

**Table 6:** Results from the statistical tests performed on RC values for overall and individual algorithm data

	Mann–Whitney *U* test (*P* value)	Spearman correlation test (*ρ*, *P* value)	Bland–Altman plot [d ± 1.96SD (%)]
RC_mean_	0.611	0.974, *P* value < 0.001	2.1 ± 16.9%
OSEM RC_mean_	0.599	0.979, *P* value < 0.001	4.4 ± 14.5%
BPL RC_mean_	0.650	0.960, *P* value < 0.001	− 0.32 ± 18.1%
RC_max_	0.720	0.974, *P* value < 0.001	3.3 ± 22.3%
OSEM RC_max_	0.578	0.977, *P* value < 0.001	5.9 ± 22.2%
BPL RC_max_	0.853	0.967, *P* value < 0.001	0.77 ± 21.6%

## Discussion

As mentioned in the background section, hybrid method using physical data and analytical simulation can be used as an alternative solution for the evaluation of PET scanners, compared to pure simulated data or experimental procedures.

A key assumption of this kind of method is that the forward projection model needs to be able to closely represent the relationship between image and raw data domains. While this method can be used to evaluate the system response under this assumption, any imperfections in the forward projection model would be blended into the generated data and would not be detected by the current approach. For this reason, the current method would not be able to answer the question of whether the forward projection model is exact or not but can be used to compare the results in different activity and noise combinations with several replications. Another method to compare with the current approach is Monte Carlo simulation [8, 9]. While the Monte Carlo simulation seems to be able to faithfully simulate the data with a given system model, the system model is never perfect. In this case, Monte Carlo simulated data may suffer from the imperfections of the simulated models, including inaccurate crystal chemical compositions, electronic design, single-to-coincidence stream, etc. Therefore, a Monte Carlo approach may not be a perfect solution for assisting system evaluation, and the proposed approach can be a good fit to complete the system evaluation along with physical phantom measurements.

In this study, we aimed to introduce a hybrid simulation method and to evaluate its accuracy in multiple scenarios in comparison with equivalent physical experiments.

Based on real images and using a model of the scanner, the method generated images whose visual rendering and visualization of objects are similar to experimental images. Discrepancies observed in the visual comparison occurred for target presenting challenging size and contrast in terms of detection for the device. It is the limit of the method which shows slight deviation from the physical images when the limits in performance of the system are reached. Nevertheless, given this specificity and weakness of these differences, we considered them negligible for our study and the final clinical implementation and assessed the equivalence between the two datasets. From the quantitative analyses, we were able to verify that the experimental and simulated data were comparable, correlated, with differences normally distributed. Both algorithms showed no statistical differences and close results in terms of correlations and limits of agreement. Although, we could highlight higher mean differences for OSEM RC_mean_ and RC_max_ due to the noise reduction induced by the regularization algorithm. Indeed, BPL applies activity-dependent smoothing and suppresses image noise in low-activity regions [15]. In our case, the cold spheres from the original images represented the insertion locations. Hence, it resulted in a deviation for the mean difference between BPL (less than 1%) and OSEM (within 6%) considering RC_mean_ and RC_max_, which could be explained by the study setting and the choice of not having activity inside the spheres for the original acquisition.

In this study, we demonstrated the reliability of the method applied under specific experimental conditions through the insertion of synthetic spheres and their comparison with equivalent experimental data defined as reference. We showed that we were able to simulate realistic visual and quantitative results compared to experimental data even under challenging situations such as small and low contrast targets. Based on computer programs developed by the PET manufacturer, the method uses the same processes available on the physical systems. In contrast to some simulation studies [11–13], the method offers all available features of the physical system like PSF or TOF implementation. Files generated during the initial acquisition and reconstruction are used to generate new datasets inserting virtual information to obtain a practical render of the exam. These synthetic datasets are useful for qualitative and quantitative assessment of system performance as they combine real backgrounds with inserted objects of known size, activity and location. From a single dataset, it allows to generate as many configurations as needed without requiring access to the scanner, which may be limited in terms of device and radiotracer availability. In addition, it can be applied directly to clinical data in order to evaluate impacts of acquisition and reconstruction parameter on patient examination [14, 15]. We intend to employ this method to support the physical and clinical evaluation phase of a new PET–CT device as part of the collaborative research partnership with the manufacturer.

Our final objective is to extend our simulation method to patient data in order to evaluate the impact of small lesions with low activity on clinical images while keeping control over the inserted object (location, size, activity, shape and pattern).

Computation times for generating sinograms were significantly shorter than reconstruction times. With TOF, the simulation duration was about 15 min, and without TOF it decreased to nearly 2 min. Currently, reconstruction times are about 2 h for the OSEM algorithm and 6 h for the BPL algorithm for a single bed position. For the same reconstruction parameters without TOF, we observed reconstruction times of approximately 25 min for the OSEM algorithm and around 3 h for the BPL algorithm. It is possible to parallelize the reconstructions of different bed positions (for example, a clinical examination) and thus reduce the reconstruction time to a single bed position. We are working on exporting the modified raw data to the PET console to drastically shorten the reconstruction process. Hence, it will be possible to perform the simulation into raw data on the workstation and remigrate them afterward to the physical PET scanner and generate reconstructed images in minutes.

One of the underlying limitations in the model-based projection method is that inserted information does not add scatter and random coincidences in the resulting sinogram. Hence, the modified data include only the original random and scattered coincidences. In this study, given the sizes and activities present in the spheres, we assumed that their impacts were negligible.

## Conclusion

We introduced and evaluated a hybrid simulation method using synthetic spheres inserted into acquired raw data prior to their reconstruction. The insertions can be fully controlled and provide opportunities to evaluate medical imaging functions and image processing techniques.

In the context of a collaborative research partnership, this study is a first step in using this method for the performance evaluation of the next generation of PET scanners. It will then be extended to more complex phantom models for validation and patient data to create a scalable ground truth and guide more efficiently the clinical practice.

## Supplementary Information

**Additional file 1.** Schematic illustration of the modeling phase for the sphere mask generation.

## Data Availability

The datasets used and/or analyzed during the current study are available from the corresponding author on reasonable request.

## Abbreviations

PET: Positron emission tomography
CT: Computed tomography
CTAC: Computed tomography attenuation correction
NEMA: National Electrical Manufacturers Association
IEC: International Electrotechnical Commission
OSEM: Ordered subset expectation maximization
BPL: Bayesian penalized likelihood
SBR: Sphere-to-background ratio
TOF: Time-of-flight
FOV: Field of view
IQ: Image quality
^18^F: Fluorine-18
PSF: Point spread function
VOI: Volume-of-Interest
AC: Activity concentration
AC_mean_: Mean activity concentration
AC_max_: Maximum activity concentration
RC: Recovery coefficient
RC_mean_: Mean recovery coefficient
RC_max_: Maximum recovery coefficient
SD: Standard deviation
