# Supplementary material for: Evaluation of a method based on synthetic data inserted into raw data prior to reconstruction for the assessment of PET scanners
Source: EJNMMI Phys. 2022 Oct 1;9:68. doi: 10.1186/s40658-022-00496-6 (PMC9526779; doi:10.1186/s40658-022-00496-6)
Supplement: Supplementary file 1 — Additional file 1. Schematic illustration of the modeling phase for the sphere mask generation. [file 40658_2022_496_MOESM1_ESM.docx]

*Additional file 1*

*
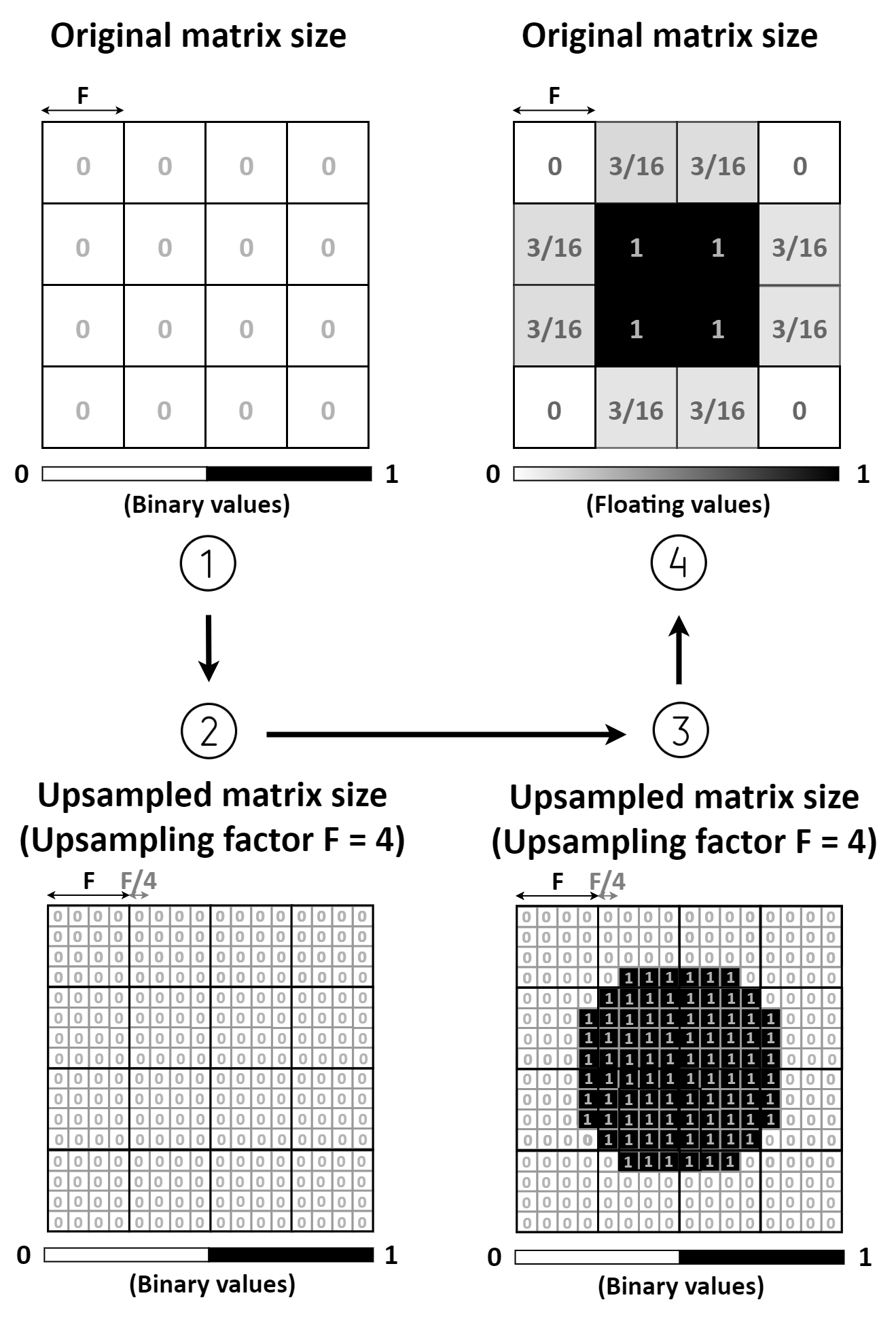
*

*Figure S1: Schematic illustration of the modeling phase for the sphere mask generation.*
